# Cooperative Role of Carbonic Anhydrase IX/XII in Driving Tumor Invasion and Metastasis: A Novel Targeted Therapeutic Strategy

**DOI:** 10.3390/cells14100693

**Published:** 2025-05-11

**Authors:** Hanyu Yang, Rui Chen, Xiang Zheng, Yufan Luo, Mingxuan Yao, Famin Ke, Xiurong Guo, Xiaoyan Liu, Qiuyu Liu

**Affiliations:** 1 School of Pharmacy, Southwest Medical University, Luzhou 646000, China20240599120129@stu.swmu.edu.cn (R.C.);; 2 Department of Clinical Medicine, School of Clinical Medicine, Southwest Medical University, Luzhou 646000, China; 3 Laboratory of Metabolism, Center for Cancer Research, National Institutes of Health, Bethesda, MD 20892, USA

**Keywords:** carbonic anhydrase IX (CA IX), carbonic anhydrase XII (CA XII), invasion, tumor metastasis, therapy

## Abstract

Cancer invasion and metastasis are critical factors that influence patient prognosis. Carbonic anhydrase IX (CA IX) and carbonic anhydrase XII (CA XII) are key regulators of hypoxia and pH homeostasis in the tumor microenvironment (TME). It has been verified that both CA IX and CA XII play significant roles in promoting tumor metastasis in recent years, but most of the literature tends to treat them as separate entities rather than exploring their synergistic effects. This review provides a comprehensive overview of the roles of CA IX and CA XII in tumor invasion and metastasis, along with their clinical applications, including their spatial distribution characteristics, molecular mechanisms that facilitate tumor metastasis, and their potential for clinical translation. Moreover, this review incorporates the classical tumor core–invasive front model to propose a metabolic coupling model of CA IX and CA XII, offering a fresh perspective on precision therapies that target tumor metabolism. By emphasizing the metabolic coupling between these two molecules, this review offers new insights distinct from previous studies and highlights the clinical therapeutic potential of simultaneously targeting both during treatment. It sheds new light on future research and clinical applications, aiming to enhance the prognosis of cancer patients through innovative therapeutic strategies.

## 1. Introduction

Cancer is a disease that remains difficult to cure, and its complexity and heterogeneity pose significant challenges to current therapeutic strategies. In recent years, the global cancer burden has been escalating, and it is projected that cancer will soon become the leading cause of premature mortality worldwide. According to GLOBOCAN statistics, the incidence of cancer is rising rapidly, with new global cases expected to reach 35 million by 2050—an increase of 77% compared to 2022. This trend is particularly pronounced in countries with a low Human Development Index (HDI), where the number of new cases is projected to double [1,2]. At present, the outlook for cancer treatment remains challenging, necessitating in-depth investigations into the mechanisms underlying tumor initiation and progression, as well as the continuous exploration of novel therapeutic approaches and strategies. Tumor invasion and metastasis are central topics in cancer research as they are directly linked to tumor malignancy and patient prognosis. Specifically, the invasive and metastatic potential of tumors serves as a critical criterion for prognostic evaluation. Highly invasive tumors are often associated with increased recurrence rates and a heightened risk of metastasis, ultimately affecting patient survival and quality of life. Therefore, a comprehensive discussion of tumor invasion and metastasis becomes a crucial aspect of cancer research [3,4].

CO_2_ is a simple molecule that is widely present in nature. For living organisms, CO_2_ serves as a stable and abundant carbon source. Under natural conditions, CO_2_ reacts with water to form unstable carbonic acid, which subsequently dissociates within biological systems into bicarbonate and hydrogen ions (CO_2_ + H_2_O ⇋ HCO_3_^−^ + H^+^). Through this process, the carbon atoms in CO_2_ become bioavailable and enter metabolic cycles. However, the reaction between CO_2_ and water occurs at an inherently low efficiency under natural conditions, limiting the ability of organisms to utilize this abundant environmental carbon source. To overcome this challenge, organisms have evolved an enzyme that catalyzes the reversible hydration of CO_2_, known as carbonic anhydrase, which was first discovered in 1933 [5]. To date, several families of carbonic anhydrase enzymes have been identified, including α-, β-, γ-, δ-, ζ-, η-, θ-, and ι-CAs. These enzyme families are genetically independent of each other [6]. Most carbonic anhydrases (α-, β-, γ-, δ-, ζ-, η-, and θ-CAs) are metalloenzymes, and their cation properties are variable, exhibiting enhanced activity upon binding with Zn(II) [7].

In the human body, carbonic anhydrase is primarily of the α-CA type, with several isoforms present [8]. These isoforms vary in their distribution, quantity, and function across different cells, tissues, and organs in the human body. As a result, there are significant differences in the structure, pharmacological functions, and clinical effects of their inhibitors [9]. They have widespread applications both in traditional therapeutic fields and in the development of novel drug targets for conventional medications [10,11,12,13,14]. The existing literature reports that CA IX and CA XII have profound effects on various physiological activities of tumor cells, although the actions they produce are not entirely the same [15,16,17,18]. However, to date, discussions on the combined effects of these two enzymes on tumor physiological activities remain scarce. Only a few studies have suggested that CA IX and CA XII play a role in the invasion and metastasis of cancer cells [19]. It is noteworthy that the specific metabolic coupling relationship between CA IX and CA XII has been scarcely explored, although the metabolic compartmentalization characteristics of them have been preliminarily studied. Referring to the classical “core–invasive front” model in tumor research, CA IX exhibits high expression in the tumor core, while CA XII is highly expressed at the invasive front. They are not isolated within different compartments; rather, they are interconnected through a specific metabolic mechanism, which jointly influences tumor metabolism and invasive metastatic behavior, providing a new perspective for research on tumor metabolic metastasis.

This review provides a comprehensive summary of the roles of CA IX and CA XII in tumors, with a particular focus on the unique effects they exert on various physiological behaviors of tumor cells. CA IX and CA XII play crucial roles in regulating intracellular pH and promoting tumor cell invasion and metastasis, and the review delves deeply into the mechanisms through which these effects occur. Furthermore, the review discusses the clinical application prospects of CA IX and CA XII, including the current development status of enzyme inhibitors, antibody drugs, and combination therapies targeting these molecules. This review systematically summarizes the synergistic effects of CA IX and CA XII in tumor metastasis for the first time and proposes a metabolic coupling model based on the “core-invasive front” model. It provides new perspectives for understanding tumor metabolic adaptability and changes in the tumor microenvironment (TME), as well as opening new avenues for targeted therapeutic strategies.

## 2. CA IX and CA XII in Cancer Metastasis

Human carbonic anhydrases (h-CA) consist of sixteen α-CA isoenzymes, which exhibit differences in tissue distribution, kinetic properties, and physiological functions [20]. It is currently widely accepted that CA IX and CA XII (among the various isoforms of h-CA) are highly associated with the development of cancer. Specifically, the membrane-bound carbonic anhydrase isoform CA IX is significantly overexpressed in tumor cells compared to normal cells [21,22]. Furthermore, enzymatic assays indicate that CA IX exhibits higher catalytic activity in low pH environments, which is consistent with the hypoxic and acidic TME resulting from metabolic reprogramming in tumor cells [23]. In addition to its synergistic effect with CA IX, CA XII may also exhibit strong correlations with certain components of the TME, such as acidic and hypoxic conditions, as well as the ECM. By influencing these components, CA XII enables tumor cells to gain enhanced survival, increased invasiveness, and immune evasion. Growing evidence suggests that these two carbonic anhydrases may interact with each other and play crucial roles in regulating the TME and promoting tumor metastasis. Therefore, a deeper understanding of the roles of CA IX and CA XII in tumors can provide new insights and inspiration for research on the mechanisms of tumor metastasis and has gradually become a growing area of focus in recent years.

### 2.1. CA IX in Cancer Metastasis

The expression of CA IX is restricted in normal tissues, where it is only expressed in a few tissues, primarily in the gastrointestinal tract [24,25]. However, its expression is significantly upregulated in tumor cells, where it serves as a hypoxic marker in various solid tumors [26]. CA IX has multiple roles in tumor cells. It is widely believed that CA IX, as one of the key molecules in the HIF transcriptional regulation network, plays an important role in maintaining pH in tumor cells, which is significant for maintaining cellular metabolic activity and enhancing cell survival. It is currently believed that PKC is likely to play an important role in this process [27]. It is well known that changes in pH in the TME have an important impact on tumor metastasis, but the role of CA IX in this process has rarely been mentioned. It suggests that CA IX may have a currently underexplored mechanism that contributes to tumor metastasis in addition to its traditional role in regulating pH, providing a new perspective for research on tumor metastasis. Additionally, exosomes, as important mediators of intercellular communication, are closely related to tumor invasion and metastasis. Notably, the presence of CA IX alters certain characteristics of ordinary exosomes, causing cells to exhibit markedly different behaviors, which provides a new entry point for studying the role of CA IX in tumor metastasis. The pH-regulating function of CA IX is closely related to the hypoxic environment of tumor cells. When the oxygen concentration in the microenvironment decreases, the HIF-α subunit remains stable by escaping VHL-mediated ubiquitination degradation, leading to the accumulation of HIF-α in cells [28]. In the analysis of over 400 cases of clear cell renal cell carcinoma (ccRCC) from the TCGA database, approximately 80% of cases exhibit VHL gene mutations or promoter methylation. This alteration stabilizes HIF-α, resulting in the sustained high expression of downstream hypoxia-related genes, such as CA IX, one of the downstream genes regulated by HIF-1α [26,29,30,31]. Under hypoxic conditions, HIF-1α binds to the hypoxia response elements (HRE) in the promoter region of CA IX, activating its transcription. As one of the key molecules in the HIF transcriptional regulation network [32,33,34,35], CA IX responds to physiological stresses such as low oxygen concentration (hypoxia) and the accumulation of acidic products (pH) by catalyzing the CO₂ hydration reaction. Specifically, CA IX sequesters the excess CO₂ produced by aerobic glycolysis and participates in the process of proton (H^+^) extrusion, thereby maintaining the stability of intracellular pH [36]. The CO₂ generated in this process serves as a substrate for carboxylation reactions and enters fatty acid synthesis mediated by acetyl-CoA carboxylase (ACC), which contributes to replenishing oxaloacetate in the tricarboxylic acid (TCA) cycle and thus maintains aspartate metabolism, helping to resolve the inhibition of the electron transport chain caused by hypoxia [37]. Therefore, CA IX maintains the stability of the TME and metabolic balance through various mechanisms, serving as a crucial molecular foundation for cellular adaptation to harsh conditions such as hypoxia. On the other hand, some studies suggest that CA IX also plays an important role in promoting tumor cell metastasis. Research indicates that CA IX drives extracellular matrix (ECM) degradation and remodeling through its dual roles in acidifying the microenvironment and signal transduction, which enhances tumor invasion and metastasis through exosome-mediated angiogenesis, exhibiting organ-specific patterns during the metastasis process. Its influence on tumor metastasis is exerted through the combined actions of multiple molecules at multiple levels.

**Figure 1:**
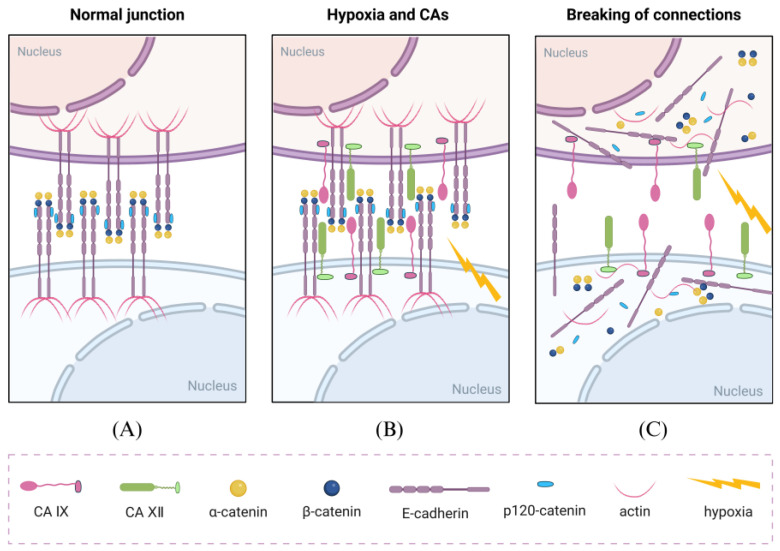
Disruptive effects of CA IX and CA XII on cell adhesion. (**A**) Under normal conditions, cell adhesion-related proteins (such as E-cadherin and β-catenin) are normally expressed, forming tight intercellular junctions that inhibit cell migration and motility. (**B**) Under hypoxic conditions, the expression of CA IX and CA XII is upregulated in cells, and these proteins can interact with E-cadherin. (**C**) Under the influence of CAs, intercellular adhesion is disrupted, greatly promoting the invasion and metastasis of tumor cells. Created with BioRender.com.

Changes in pH have a significant impact on tumor metastasis. Under acidic conditions, tumor cells may splice certain key proteins, and these protein alterations contribute to increased aggressiveness, enabling tumor cells to breach the surrounding ECM. It suggests that acidity may trigger the alternative splicing of specific genes and participate in ECM degradation, thereby facilitating tumor cell metastasis [38]. CA IX plays a role in regulating pH changes, suggesting that it is highly likely to influence cell invasion and metastasis through this mechanism. For instance, when the ECM becomes more acidic (pH 6.0–6.5), pro-matrix metalloproteinases (pro-MMPs) undergo conformational changes that expose their active sites, thereby disrupting intercellular adhesion, enhancing tumor cell invasiveness, and promoting tumor metastasis [39,40]. Specifically, membrane-type matrix metalloproteinase 1 (MT1-MMP) cleaves pro-MMP-2 through its catalytic domain, converting it into active MMP-2 (64 kDa), which degrades components of the ECM, including collagen and fibronectin, and participates in cytoskeletal remodeling, promoting the formation of pseudopodia, ultimately enhancing the invasiveness of tumor cells [41,42]. Additionally, CA IX can reduce cell adhesion and enhance invasiveness through multiple mechanisms, including interacting with β-catenin, influencing the expression of integrins, focal adhesion kinase (FAK), paxillin, cell adhesion-related proteins, as well as regulating intracellular metastasis-associated signaling pathways [43,44] (Figure 1).

**Figure 2:**
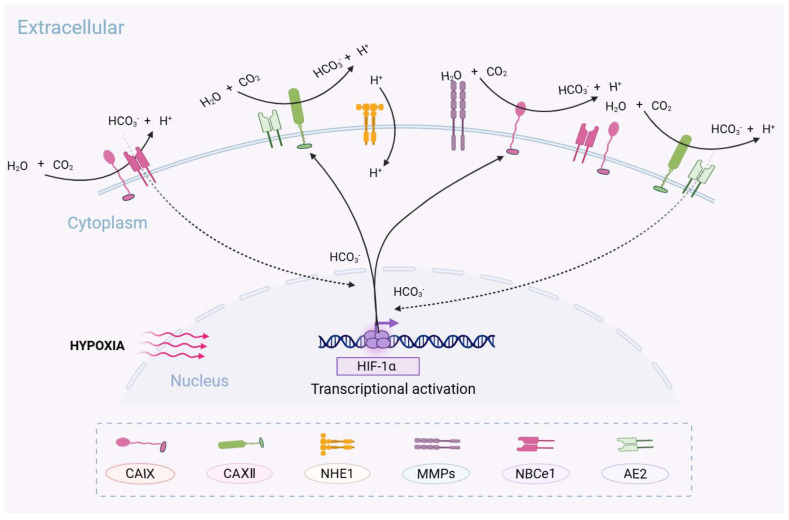
CA IX and CA XII are closely associated with specific proteins in hypoxia-induced cell invasion. During hypoxia-induced invasion, CA IX and CA XII can be coupled with specific proteins to exert synergistic effects. CA IX and CA XII can associate with membrane ion transporters NBCe1 and AE2, facilitating the influx of HCO_3_^−^ produced by CA-catalyzed hydration of CO_2_, thereby maintaining intracellular pH homeostasis and influencing gene expression in concert with hypoxia. CA IX can also colocalize with matrix metalloproteinases (MMPs) and sustain their activity through H⁺ generated by CO_2_ hydration. It enhances the activity of other MMPs (such as MMP2, MMP3, and MMP9), ultimately affecting the composition of the extracellular matrix. Created with BioRender.com.

The expression and activity of CA IX also play a crucial role in maintaining the mesenchymal phenotype (EMT) of breast cancer stem cells (CSCs). Persistent high expression of CA IX promotes EMT, which is a process essential for tumor cells to acquire migratory and invasive capabilities and a prerequisite for metastasis, ultimately leading to the invasiveness of CSCs [45]. This regulation facilitates the further enhancement of aerobic glycolysis, exacerbating microenvironmental acidification and promoting resistance to cellular stress [34,39]. Previous research has suggested that CA IX may also directly participate in intercellular signaling through its extracellular domain (e.g., activation of the EGFR/PI3K pathway) or influence cell adhesion and differentiation, which is independent of its enzymatic activity contributing to tumor progression [46,47]. On the other hand, CA IX may also interact with other pH-regulating proteins on the cell membrane. NHE1 (Na^+^/H^+^ exchanger 1) is an ion transport protein that is crucial in tumor cell migration, invasion, proliferation, and chemoresistance. It can indirectly promote tumor cell migration by modulating cytoskeletal dynamics, acidifying the cell surface, and enhancing MMP activity [48]. There are currently no direct studies on the interaction between NHE1 and CA IX; however, both are directly involved in proton transfer in tumor cells, and protein expression analyses in various tumor samples suggest a potential yet unexplored connection between them. Finally, CA IX exhibits organ-specific patterns in tumor metastasis. During bone metastasis, CA IX promotes the degradation of type I collagen through MMP-13, resulting in the release of TGF-β and IGF-1, which in turn activate osteoclasts [49]. During brain metastasis, the CA IX/MMP-2 axis disrupts the tight junction proteins of the blood–brain barrier and the ECM, facilitating the intracranial migration of tumor cells [50] (Figure 2).

These findings indicate that the impact of CA IX on tumor metastasis relies on the cooperative action of multiple molecules and layers. The regulation of tumor metastasis by CA IX is subtype-specific and exhibits spatiotemporal dynamics. The multilayered ECM remodeling mechanism positions CA IX as a central hub linking metabolic adaptation with physical microenvironment remodeling.

**Figure 3:**
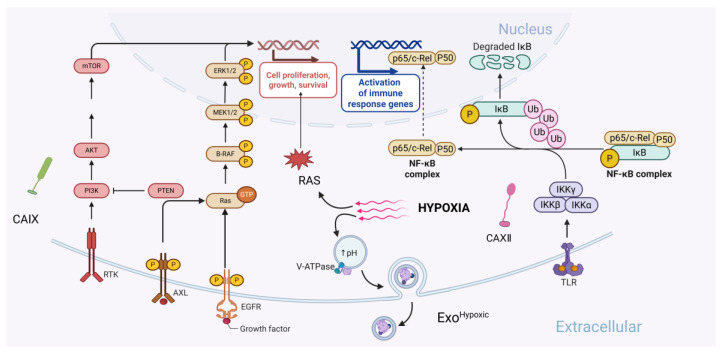
Overview of carbonic anhydrases-associated signaling pathways in tumor cells. CA IX can influence signal transduction through the EGFR/PI3K pathway, enhancing pathway activity and thereby regulating the expression of proteins related to tumor cell growth and survival. CA XII modulates the activity of NF-κB-related proteins, primarily by promoting the ubiquitin-mediated degradation of IκB, allowing the p65/cRel–P50 complex to translocate into the nucleus and exert its effects, thereby impacting tumor-associated immune responses and therapeutic outcomes. Acidic and hypoxic conditions regulate tumor physiology mainly through RAS and Exo pathways. RAS signaling enhances the expression of genes associated with cell growth and survival, while exosomes under hypoxia and acidic conditions exhibit distinct features compared to those in other environments, including smaller size, higher quantity, elevated protein expression, and greater functional activity. These combined signals promote tumor cell growth, survival, and immune evasion. Created with BioRender.com.

Additionally, as an important medium of intercellular communication, exosomes provide a novel perspective on the role of CA IX in tumor metastasis, distinct from traditional metabolic pathways. Exosomes, a type of extracellular vesicle (EV), are released into the extracellular space after the fusion of multivesicular bodies (MVBs) with the cell membrane. Exosomes have a lipid bilayer structure and are capable of carrying and transmitting a variety of bioactive molecules, including proteins, lipids, mRNA, and miRNA. Current research on exosomes focuses on their roles in cancer progression, metastasis, and immunity. Exosomes hold significant promise as cancer biomarkers and are being developed as targeted cancer therapeutics, showing promising clinical application potential [51,52,53]. Although research on CA IX exosomes is still in its early stages, significant progress has been made. In the acidic TME, the characteristics of exosomes undergo significant changes compared to other environments. Under acidic conditions in the LNCaP cell line (human prostate cancer cell line), the release of exosomes is higher than those in normal cells. Both the expression and activity of CA IX in the exosomes are significantly increased, with notable changes in the expression pattern [22]. Under hypoxic conditions, exosomes secreted by the LNCaP cell line (Exo^Hypoxic^) have a smaller average size and contain higher levels of tetraspanin, heat shock proteins, and Annexin II. It has been shown that Exo^Hypoxic^ significantly enhances the invasive and migratory capacity of LNCaP cells, specifically manifested due to the following reasons: (i) Exo^Hypoxic^ promotes the formation of prostate cancer spheroids, enhancing their stem cell properties; (ii) Exo^Hypoxic^ increases the expression of α-SMA in prostate stromal cells and induces the conversion of these stromal cells into cancer-associated fibroblasts (CAFs); and (iii) Exo^Hypoxic^ exhibits higher metalloproteinase activity and targets E-cadherin and β-catenin, disrupting intercellular adhesion, and promoting the invasion and migration of the cells [54]. The expression of CA IX is significantly elevated in renal cell carcinoma (RCC) cells, which form exosomes containing CA IX protein through MVBs, which can be taken up by human umbilical vein endothelial cells (HUVECs). This process significantly enhances the migratory capacity and angiogenic ability of HUVECs, promoting angiogenesis and thereby increasing the tumor invasiveness and metastatic potential [55], which reveals its core role in the formation of the pre-metastatic niche during tumor invasion and metastasis [52,56,57]. Its unique dual regulation of enzyme activity and signal transduction, along with its organ-specific characteristics, strongly demonstrates its potential as a therapeutic target in the direction of precision treatment for tumors (Figure 3).

### 2.2. CA XII in CANCER Metastasis

CA XII is another member of the carbonic anhydrase family, and it is highly expressed in tumor tissues and tumor-associated immune cells, especially in tumor-infiltrating monocytes and macrophages. CA XII is associated with tumor progression and poor prognosis in patients through the production of chemokines and the regulation of intracellular signaling in tumor cells, and it has gradually gained attention in tumor research becoming a new research perspective [58].

Recent studies show that CA XII exhibits a unique expression pattern in cancers—it is not directly expressed in tumor cells but specifically accumulates in tumor-associated macrophages (TAMs), particularly in the CD206^+^/CD204^+^ M2-like macrophages in human hepatocellular carcinoma (HCC). It suggests that CA XII may play a crucial role in the survival and function of the M2-like macrophages in HCC tumor tissues [59]. Pathological studies have shown that the upregulation of CA XII expression is significantly correlated with tumor stage, metastasis rate, and poor prognosis, suggesting that it could serve as a novel biomarker for prognosis evaluation. Mechanistic studies indicate that CA XII helps maintain the pH within macrophages, protecting them from damage in acidic environments, thus enabling macrophages to survive and function under such unfavorable conditions. Furthermore, macrophages with high CA XII expression induce the production of C-C motif chemokine ligand 8 (CCL8) through the p38 signaling pathway. CCL8 is an important chemokine that promotes EMT in tumor cells, enhancing their migration and invasion capabilities. Animal experiments have demonstrated that the expression level of CA XII is positively correlated with tumor size and metastasis, and treatment with CA XII inhibitors resulted in reduced tumor volume and lower invasiveness in mouse models, confirming its potential as a therapeutic target [59,60,61].

CA XII is strongly associated with NF-κB, and it can influence tumor cell apoptosis and drug treatment efficacy by regulating the activity of NF-κB-related proteins. In pancreatic adenocarcinoma (PAAD), the expression of CA XII is significantly downregulated and associated with a poorer prognosis. Results show that overexpression of CA XII significantly inhibits the proliferation, invasion, and migration of PAAD cells, meanwhile, inducing cell cycle arrest at the phase of G0/G1 and apoptosis. In contrast, the downregulation of CA XII significantly suppresses these processes. Moreover, when CA XII is overexpressed, the level of NF-κB p65 protein decreases, and the IκBα protein increases, with both changes being significant. Conversely, when CA XII is knocked down, the opposite effects are observed. Given the close relationship between NF-κB, cell apoptosis, and invasion, CA XII is likely to influence apoptosis and metastasis by regulating NF-κB-related proteins, demonstrating its potential as a therapeutic target for cancer treatment [62]. Auranofin (AF) is a drug originally used to treat rheumatoid arthritis, due to its unique biological mechanisms, it has recently been investigated as an anti-cancer drug. Research has shown that AF exhibits good cytotoxicity against non-small cell lung cancer (NSCLC) cells and pancreatic ductal adenocarcinoma (PDAC) cells at concentrations below 1 μM, with minimal impact on normal cells, showing good selectivity and clinical application potential. Further studies have found that cancer cells with low CA XII expression are more sensitive to AF treatment, suggesting that CA XII could serve as a biomarker for predicting AF sensitivity. As mentioned above, CA XII could be closely associated with NF-κB, and it is reasonable to hypothesize that AF may exert its therapeutic effect by inhibiting NF-κB-related proteins. In cancer cells with low CA XII expression, the NF-κB signaling pathway is more active, which enhances the therapeutic effect of AF, highlighting the potential of CA XII as both a cancer treatment target and a biomarker [63]. In addition, other studies have shown that factors such as LncRNA and histamine regulate the expression of CA XII in tumors, inhibiting tumor survival, metastasis, and drug resistance [64,65]. These studies indicate that the role of CA XII in tumors is gradually gaining attention, and its potential as a therapeutic target for cancer treatment is becoming increasingly evident (Figure 3).

## 3. Synergistic Effect of CA IX and CA XII

CA IX and CA XII are involved in a closely coordinated metabolic network during tumor physiological processes; however, the evidence of direct molecular interactions between them still require further investigation. Multiple lines of evidence suggest that the interaction between CA IX and CA XII ultimately promotes tumor progression and invasion, leading to poor prognosis for patients. First, CA IX is widely overexpressed in tumor cells, catalyzing the conversion of CO_2_ to HCO_3_^−^ in hypoxic regions, neutralizing intracellular acidity, and participating in proton efflux, thus helping tumor cells adapt to the hypoxic environment. CA XII is highly expressed in M2-like macrophages in certain tumors, aiding in the maintenance of the acidic TME and helping tumors resist acid-induced damage. In general, tumor cells recruit immune cells to the tumor periphery, a process that usually promotes tumor development, including enhancing tumor proliferation, weakening anti-tumor immune responses, promoting new tumor blood vessel formation, and assisting tumor immune evasion [66]. The high expression of CA XII in macrophages can accelerate this process, further affecting the acid–base balance and immune infiltration within the TME. CA IX and CA XII can synergistically maintain the tumor survival advantage through “intracellular alkalinization (CA IX)” and “extracellular acidification buffering (CA XII)”.

It is currently widely accepted that CA IX and CA XII regulate both intracellular and extracellular pH, while hypoxia-induced extracellular acidification can in turn affect the activity of CAs, forming a feedback regulatory loop. Under hypoxic conditions, the expression of CA IX and CA XII is regulated by HIF-1, which simultaneously enhances the catalytic activity of CA IX and CA XII [23,67]. Additionally, low pH levels upregulate CA XII expression in tumor-associated macrophages, activate NF-κB, and induce CCL8 secretion [59]. It constitutes a crucial positive feedback loop: hypoxia-induced acidosis enhances CA IX/CA XII activity, which further intensifies extracellular acidification, thereby promoting extracellular matrix degradation and tumor metastasis. Importantly, the effects vary among different cell types. In tumor cells, CA IX primarily maintains an alkaline intracellular environment to support cell survival; whereas in tumor-associated macrophages, CA XII mainly ensures macrophage survival under acidic conditions and facilitates immune evasion through the release of chemokines. The cell-specific functions of these enzymes highlight the complexity of targeting them therapeutically. Therefore, careful consideration must be given to their differential expression and function across various cellular populations in designing treatment strategies aimed at these two isoforms.

Deuterium-Depleted Water (DDW) is a type of water processed through specialized techniques to significantly reduce its deuterium (^2^H) content compared to that found in natural (ordinary) water. Although not yet widely applied in clinical cancer therapy, DDW has demonstrated considerable potential. Studies have shown that DDW can influence extracellular acidification of tumor cells through multiple mechanisms, including: (a) altering cancer cell metabolic strategies to reduce lactate production and secretion; and (b) affecting V-ATPase function, thereby decreasing proton efflux and markedly improving tumor cell drug sensitivity. These properties enable DDW to inhibit tumor cell proliferation, indirectly modulate immune responses in the TME, and enhance the cytotoxicity of immune cells against tumor cells, ultimately contributing to improved patient survival [68,69]. Moreover, studies have shown that in tumors with high expression of CA IX and CA XII, the tumor cells exhibit more invasiveness compared to other types of tumors [55,70,71]. It is due to the combined effects of CA IX and CA XII. On one hand, the high expression of CA IX in tumor cells leads to a downregulation of E-cadherin expression, while the expression of N-cadherin and Vimentin is upregulated, accelerating the process of EMT [72,73]. On the other hand, CA XII-positive macrophages directly induce EMT by secreting CCL8, and recruit immune-suppressive myeloid cells to form a pro-metastatic microenvironment [59]. CA IX promotes tumor angiogenesis by inducing VEGF and other pro-angiogenic factors [74], while CA XII+ macrophages secrete CCL8, which recruits endothelial progenitor cells and synergistically enhances angiogenesis [75,76]. From another perspective, this also suggests that both have a strong ability to enhance tumor metastasis and invasion. The original pro-metastatic effect is amplified under the multifaceted synergistic actions, which significantly enhance the invasiveness and metastatic potential of the tumor.

It is worth noting that tumor cells with high expression of CA IX and CA XII can cause drug resistance [77,78,79]. The acidic environment maintained by CA IX and CA XII through multiple pathways can affect the solubility and permeability of drugs, thereby reducing the efficacy of chemotherapy drugs. Furthermore, the high expression of CA IX and CA XII can activate drug efflux pumps, such as P-gp, which expel chemotherapy drugs from the cells, decreasing the intracellular drug concentration and ultimately leading to drug resistance. In neuroblastoma, the high expression of CA IX is significantly associated with poor prognosis and high risk, as it inhibits cisplatin-induced ferroptosis, promotes cell proliferation, and reduces drug efficacy [80]. When CA IX and CA XII inhibitors are used to suppress the activity, the sensitivity of tumor cells to cisplatin significantly increases, and the treatment effect improves significantly [21]. The expression level of CA XII shows a significant positive correlation with multidrug resistance in breast cancer. CA XII primarily enhances the drug efflux capacity of tumor cells by upregulating the expression of P-gp, resulting in drug resistance [81]. Currently, there has been development of combined inhibitors targeting both P-gp and CA XII. These inhibitors are expected to reverse multidrug resistance in tumors and improve the efficacy of chemotherapy [82].

The expression of HIF-1α is positively correlated with that of CA IX in cancer, and regions of high CA IX expression are typically accompanied by an enrichment of CA XII. It suggests that CA IX and CA XII may share hypoxia-driven metabolic pathways. Based on this, it can be speculated that the HCO_3_^−^ produced by CA IX may be transmitted to TAMs through gap junctions or extracellular vesicles, serving as a substrate for the CA XII-catalyzed reaction, further regulating the extracellular pH gradient. Additionally, the H^+^ efflux mediated by CA XII may enhance the catalytic efficiency of CA IX, forming a “basic inside, acidic outside” feedback loop that promotes tumor cell invasiveness. These mechanisms collectively maintain the acidic microenvironment of tumor cells [83]. Although the correlation between HIF-1 and carbonic anhydrase expression is well-established, there is limited research on the relationship between other HIF family members and carbonic anhydrases. Under prolonged hypoxic conditions, the expression of HIF-2α in tumor cells significantly increases, and its role in tumor regulation cannot be ignored [84]. Multiple members of the HIF family may participate in the regulation of CA IX and CA XII at different time points, forming a time-dependent regulatory network, which needs further validation [35,85]. It is suggested that CA IX is associated with immune evasion in tumor cells [86]; however, there is little research on CA XII and its role in tumor immune evasion. It is worth exploring whether CA XII might contribute to immune evasion in a manner similar to CA IX, potentially promoting the tumor escape from immune system surveillance, which remains an important issue to explore further.

## 4. Clinical Significance of CA IX and CA XII

### 4.1. Compartmentalization of Metabolic Regions Coupled to Core-Invasion Fronts

In clinical practice, distinguishing between the tumor core and the invasive front of the tumor is important [87]. It primarily divides tumors into two functionally distinct regions that are significantly different at the transcriptomic and epigenetic levels: the relatively stable core region and the highly active invasive front region. Cells in the core region proliferate slowly, typically existing in a hypoxic and highly acidic microenvironment. In contrast, cells at the invasive front are more active, with stronger migratory and invasive abilities, exhibiting characteristics of EMT, and are more prone to metastasis [87,88].

Studies have found that the distribution of CA IX and CA XII aligns with the “core-invasive front” division of tumors (Table 1). In the core region, HIF-1α activates the expression of CA IX, acidifying the microenvironment. CA IX and monocarboxylate transporter 4 (MCT4) colocalize, pumping lactate produced by glycolysis out of the cells, causing lactate accumulation and forming a “lactate pool”, which enhances the Warburg effect [35,89,90]. Additionally, CA IX acidifies the lysosomes, promoting ECM degradation, facilitating tumor metastasis [91,92], and inhibiting immune surveillance [93,94]. At the invasive front, the TME is relatively oxygen rich. NF-κB signaling activates CA XII, maintaining a local weakly alkaline condition to support mitochondrial oxidative phosphorylation [62,63,95]. CA XII can also work in conjunction with MCT1 to transport lactate released from the core region to the invasive front cells. Under the action of lactate dehydrogenase B (LDHB), lactate is converted into pyruvate, which enters the TCA cycle, enabling efficient utilization of carbon sources [91]. On the other hand, lactate can promote the EMT of invasive front cells through epigenetic regulation [96]. The transfer of H^+^ generates a unique inter-regional proton concentration gradient in the exchange of substances between the core region and the invasive front [36,67], forming a “proton relay” model of metabolic coupling between CA IX and CA XII, demonstrating continuity in metabolic space.

The metabolic coupling between CA IX and CA XII has profound significance for tumor development. Firstly, this process promotes tumor invasion and metastasis. On one hand, the acidic microenvironment of the core region activates MMPs, facilitating ECM protein degradation, providing physical pathways for tumor invasion. On the other hand, the EMT occurring in the invasive front cells reduces cell adhesion, enhances metastatic ability, and paves the way for tumor metastasis. Furthermore, this metabolic coupling also leads to tumor resistance to treatment. Although directly inhibiting CA IX can reduce the acidity of the core region, the invasive front can resist this change through the CA XII-MCT1 axis, reducing drug efficacy. Drugs, such as 5-fluorouracil (5-FU), are protonated in the acidic core region, making it difficult to enter the cells. Meanwhile, cells in the invasive front using oxidative phosphorylation (OXPHOS) can upregulate ABC transporters to accelerate drug efflux [98,99], further promoting tumor drug resistance. Based on the above characteristics, the drugs currently being developed clinically are mostly dual-target drugs that co-inhibit CA IX and CA XII and have shown good therapeutic effects in cancers [100,101].

**Figure 4:**
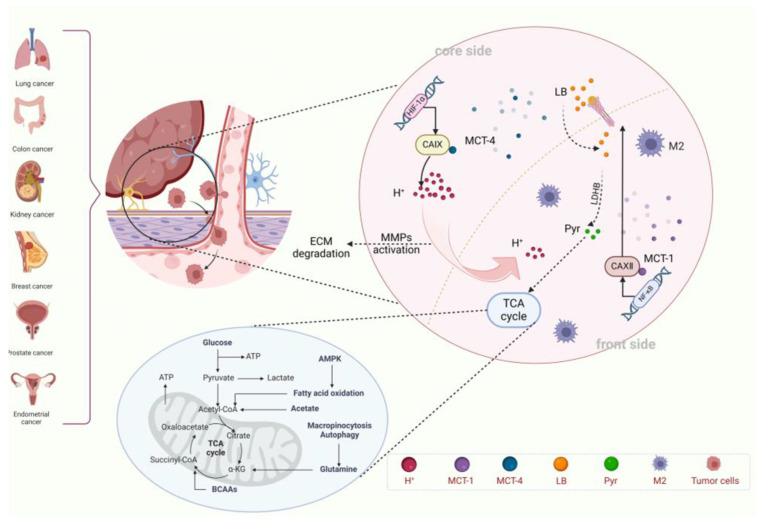
Model of metabolic regions coupled to core-invasion fronts. Tumors comprise two metabolically distinct compartments: the tumor core and the invasive front, with CA IX and CA XII expression patterns closely aligning with this spatial division. CA IX is highly expressed in the tumor core, facilitating lactate export via MCT4, forming a “lactate pool” that promotes the Warburg effect. In contrast, CA XII is highly expressed at the invasive front, working in concert with MCT1 to convert lactate into pyruvate, which enters the TCA cycle to support oxidative phosphorylation. A “proton relay” is established through lactate shuttling and proton gradient formation between these zones, promoting tumor invasion, metastasis, and resistance to therapy. Created with BioRender.com.

Although the CA IX/CA XII metabolic coupling model explains phenomena in the acidification mechanism of the TME, there are still many issues that need to be addressed. First, there is a significant discrepancy regarding the boundary of functional redundancy and compensatory effects between the two carbonic anhydrases. Current molecular mechanism studies on the association between CA IX and CA XII expression have not fully elucidated this relationship. Although a small number of preclinical model studies show that the knockout of CA IX can induce upregulation of CA XII expression [102], the universality of its compensatory effect remains unclarified. For example, the deletion of VHL leads to sustained activation of HIF-2α in ccRCC, which may serve as the molecular basis for inhibiting the compensatory role of CA XII [103,104]. However, the compensatory role of CA XII is not significant in breast cancer, suggesting that the compensatory mechanism may be dependent on the tumor type. Additionally, CA XII, as an independent enzyme, may have a pro-metastatic effect under hypoxic microenvironment that is independent of CA IX, a topic that has rarely been discussed and needs clarification regarding the functional boundary between CA IX and CA XII. Furthermore, the traditional “core-invasion front” spatial distribution model faces challenges. The conventional model suggests that CA IX is mainly enriched in the tumor core, while CA XII is localized at the invasion front. However, there may be a mixed cell population of CA IX+/CA XII+ at the invasion front in certain special tumors, such as gliomas [105]. It implies that the “core-invasion front” model of CA may have tissue-specific characteristics, and its spatial expression pattern needs to be validated in more tumor models (Figure 4).

### 4.2. Clinical Application of CA IX and CA XII

CA IX is considered a widely used marker in tumor therapy, due to its unique expression mechanism and its important role in tumor physiology, especially in evaluating the degree of tumor hypoxia and invasiveness. Similarly, CA XII, which is closely linked to CA IX, also holds prognostic significance for tumors. Evidence suggests that both CA IX and CA XII are highly expressed in cancers and are significantly associated with their invasiveness and poor prognosis [70,106,107,108]. For example, in ccRCC, CA IX is highly expressed, and its expression level is closely associated with tumor malignancy and patient prognosis, having already been established as a key diagnostic biomarker with significant clinical value [109]. Although CA IX is not currently used as a critical diagnostic marker in the clinical management of breast cancer, studies have confirmed that CA IX expression is associated with worse recurrence-free survival and overall survival in invasive breast cancer, suggesting important potential clinical significance and highlighting the need for further evaluation of its prognostic value in breast cancer [110]. Moreover, CA IX expression has also been observed in brain tumors (such as astrocytoma and ependymoma), lung cancer, ovarian cancer, and pancreatic cancer. Although research in these tumors is relatively limited, available evidence similarly indicates that CA IX expression is significantly correlated with poor prognosis and may contribute to tumor growth and metastasis [111,112,113,114].

Given the crucial roles of CA IX and CA XII in tumor progression, detecting their expression levels holds significant clinical diagnostic value. Currently, mainstream detection methods for their expression include RT-PCR, enzyme-linked immunosorbent assay (ELISA), immunohistochemistry, and Western blotting, however, these approaches present several limitations. For instance, RT-PCR relies on the concentration of mRNA in serum, and improper handling that leads to mRNA degradation may result in biased outcomes; ELISA is prone to interference from other proteins or antibodies in the serum; and immunohistochemistry is highly dependent on antibody specificity, and insufficient specificity may lead to false-positive or false-negative results. Most importantly, commonly used clinical detection methods fail to capture the dynamic changes in CA IX and CA XII protein expression and require the collection of serum or tissue samples from patients, which can negatively impact patient compliance. At present, specific molecular tracers targeting CA IX and CA XII have demonstrated potential in addressing this issue and may significantly improve their clinical detection.

In recent years, research on the clinical applications of CA IX has developed rapidly (Table 2). Girentuximab (Rencarex) is a monoclonal antibody drug targeting CA IX, primarily used for certain types of RCC, and has currently entered phase III clinical evaluation. Although some subgroups of patients may benefit from the treatment—such as those with a CA IX score ≥ 200 (high CA IX expression) and those under 65 years of age—where Girentuximab treatment was associated with improved disease-free survival (DFS), it did not significantly improve the overall DFS and overall survival (OS) of patients. As a result, the development of its therapeutic application has been halted [115]. Interestingly, even though the monotherapy studies on Girentuximab have been halted, its CA IX-targeting properties are still being actively explored. Currently, studies have found that when Girentuximab is combined with ferroptosis inducers or immune checkpoint inhibitors, it may have potential therapeutic efficacy. Therefore, Girentuximab still holds further research value [116,117]. Currently, the development of Girentuximab is mainly focused on disease diagnosis, and the diagnostic drug TLX250-CDx has progressed rapidly. Studies have shown that TLX250-CDx PET/CT exhibits high sensitivity and specificity in detecting ccRCC, especially for small tumors (≤4 cm or ≤2 cm), demonstrating its potential as a non-invasive diagnostic tool that can reduce unnecessary biopsies. Additionally, the drug has good tolerance, with a low incidence of adverse reactions, most of which are mild or related to postoperative conditions, and no severe toxicity [118,119]. Telix Pharmaceuticals submitted a Biologics License Application (BLA) to the U.S. FDA in December 2023. If it is approved, it will become the first targeted radioactive diagnostic drug for kidney cancer.

In 2022, a novel antibody–drug conjugate (ADC) targeting both CA IX and CA XII was first reported. This drug consists of monoclonal antibodies corresponding to CA IX and CA XII, along with a non-cleavable bifunctional linker, Sulfo-SMCC (sulfosuccinimidyl 4-(N-maleimidomethyl) cyclohexane-1-carboxylate). It is capable of simultaneously inhibiting multiple carbonic anhydrase isoenzymes that play a key role in tumor metabolism, providing a new tool for cancer treatment [120,121]. Small molecule–drug conjugates (SMDCs) are a new class of targeted therapeutic drugs that are formed by chemically linking small molecule ligands with cytotoxic drugs. These conjugates can specifically deliver the drug to tumor cells, thereby enhancing therapeutic efficacy while reducing toxicity to normal cells. AAZ^+^ is a specific ligand for CA IX, and the development of AAZ^+^ based on this ligand has already been underway, and their combination with certain drugs, such as those targeting interleukins, has shown promising therapeutic effects [122,123].

SLC-0111, a selective inhibitor targeting CA IX and CA XII, has demonstrated significant anti-tumor and anti-metastatic activity. Studies have shown that SLC-0111 exhibits notable inhibitory activity against human CA IX and CA XII in vitro, while having a weaker inhibitory effect on CA I and CA II widely distributed intracellular, indicating good selectivity. Furthermore, it has shown promising anti-tumor growth and metastasis effects in multiple studies and even enhances the sensitivity of tumor cells to traditional drugs such as cisplatin [21]. SLC-0111, as the first CA IX/XII inhibitor to enter clinical trials, not only demonstrates great application potential but also provides important insights for the development of new cancer therapies. It could play a significant role in the treatment of cancers in the future [124,125,126,127].

CA IX has also demonstrated its unique role among the new therapeutic approaches. chimeric antigen receptor T-cell therapy (CAR-T) is a novel immunotherapy method that involves genetically modifying a patient’s T cells to efficiently recognize and attack tumor cells in the patients’ body. Even though CAR-T cell therapy has achieved significant efficacy in hematologic malignancies, its application in solid tumors has been limited, and it faces the issue of “on-target” toxicity [128]. Studies have shown that targeting CA IX with CAR-T cells in metastatic RCC patients can result in “on-target” toxicity, and pre-treatment with CA IX monoclonal antibodies can effectively prevent hepatotoxicity and increase the tolerated dose of CAR-T cells. It provides a new perspective for optimizing CAR-T cell therapy in the future [129]. Moreover, oncolytic virotherapy (OAV-DEC), another novel therapy, can be combined with CA IX-targeted CAR-T therapy, leading to decreased tumor growth and the extended survival period of mice [130].

Recent studies have shown that researchers are developing novel applications for CA IX and CA XII beyond tumor-targeted therapy. Several newly developed monoclonal antibody drugs have characteristics beneficial for positron emission tomography (PET) imaging, suggesting the potential of CA IX and CA XII in imaging applications [47,121,131]. Traditional transcriptomic techniques struggle to analyze the spatial heterogeneity of CA IX and CA XII within the TME; however, spatial transcriptomics effectively solve this issue. Spatial transcriptomics capture and sequence mRNA on tissue sections while preserving its spatial location information. It allows for the identification of gene expression patterns within tissues or cells, helping us gain a deeper understanding of the potential relationships between CA IX and CA XII. Specifically, by using tools like Cottrazm, which can analyze the spatial interaction network between CA IX (tumor core distribution) and CA XII (invasive front distribution) at the tumor boundary. By combining single-cell sequencing with spatial transcriptomic data, subcellular-level gene expression profiles can be deconvoluted, exploring the co-regulation relationships between CA IX, CA XII, and non-coding RNAs, ultimately guiding patient stratification and the development of nano-targeted drugs [132,133,134,135].

**Table 2:** Drugs targeting CA IX/CA XII.

Drugs	Targets	Indications	Development Stage	Drug Type	Refs.
Girentuximab	CA IX	Clear Cell Renal Cell Carcinoma	Phase III Clinical Trial	Monoclonal Antibody	[115,118]
SLC-0111	CA IX/CA XII	Pancreatic Cancer, Multiple Solid Tumors	Phase I Clinical Trial	Small Molecule Inhibitor	[124]
177Lu-cG250	CA IX	Clear Cell Renal Cell Carcinoma, Triple-Negative Breast Cancer, etc	Phase II Clinical Trial	Diagnostic Radioisotope Conjugated Drug	[136,137]
CA IX-PMTE	CA IX	Locally advanced clear cell renal cell carcinoma	Preclinical	Bispecific T-cell engager	[138]
ITM-31 (LuCaFab)	CA XII	Malignant glioblastoma	Phase I Clinical Trial	Antibody-drug conjugate	[139]
CA IXhu-1	CA IX	Non-small cell lung cancer	Phase I Clinical Trial	Monoclonal Antibody	[47]
SLC-149	CA IX	Various tumors	Preclinical	Small Molecule Inhibitor	[140]
JS-403	CA XIICA IXMMP2	Various tumors	Preclinical	Small molecule chemical drugs	[141]
Anti-CA XII antibody	CA XII	Liver cancer, kidney cancer, etc.	In development	Monoclonal Antibody	[108]
Bispecific antibody (CA IX + CA XII)	CA IX, CA XII	Various tumors	In development	Bispecific antibody	[138]
Antibody-drug conjugate (ADC)	CA IX, CA XII	Various tumors	In development	Antibody-drug conjugate	[120]
Immune checkpoint inhibitor combination therapy	CA IX, CA XII	Various tumors	Clinical trial	Immunotherapy combination therapy	[142]

## 5. Discussion and Conclusions

The review provides a comprehensive review of the molecular mechanisms of CA IX and CA XII in tumor survival, invasion, and metastasis, as well as their clinical application prospects. Although CA IX and CA XII exhibit different spatial expression patterns in tumors, they do not exist in isolation or act independently on tumor development. Studies have found that CA IX and CA XII can synergistically act in multiple aspects, such as regulating the pH inside and outside tumor cells, promoting EMT, influencing the release and function of exosomes, and affecting tumor cell survival and metastasis. The review also explores the current development status of inhibitors, antibodies, and combination therapy strategies targeting CA IX and CA XII for clinical use. Although this review provides a systematic overview of the roles of CA IX and CA XII in tumor invasion and metastasis, as well as their clinical applications, a deeper analysis reveals several shortcomings and limitations. Despite frequently mentioning the close metabolic synergistic network between CA IX and CA XII in tumor physiological processes, direct molecular interaction studies are scarce. Most conclusions are based on indirect evidence or correlation analysis, lacking direct evidence. It results in a superficial understanding of their synergistic mechanisms, making it difficult to gain an in-depth understanding of the metabolic synergy details. On the other hand, the traditional “core-invasion front” spatial distribution model faces significant challenges, as in certain specialized tumor cells, there may exist mixed cell subpopulations of CA IX+/CA XII+ [105]. It suggests that the “core-invasion front” model is likely to have tissue specificity. Currently, most studies are based on limited samples and lack validation from large sample sizes, making it difficult to determine the applicability of the model, which limits the construction of spatial models for tumor metabolism.

It has been confirmed that CA IX and CA XII play significant roles in promoting tumor progression; however, many issues remain unresolved. There is still limited knowledge about the direct interactions between them, such as co-localization or signal pathway crosstalk, which requires further validation through protein interaction analysis. Additionally, CA XII is generally considered oncogenic, a small number of studies have reported that CA XII may have tumor-suppressive effects [143]. These contradictory conclusions suggest that our current understanding of the molecular mechanisms related to the carbonic anhydrase family in tumor cells is still quite limited, and further in-depth studies are required. At present, the research outcomes related to carbonic anhydrases in tumors are still some distance from clinical application. This is especially true for the inhibitors currently under investigation, which still need to be further optimized due to potential off-target effects and low delivery efficiency. Interestingly, researchers proposed a novel perspective in 2018, suggesting that aside from CA IX and CA XII, other isoforms such as mitochondrial CA VA/VB and some cytosolic CAs may also play a role in tumors and have potential as targets for drug development [144]. This provides a completely new perspective for the study of the CA family, which is of significant importance. It suggests that other members of the CA family may be discovered to play roles in tumors, leading to the development of more drugs targeting CA family members in the future.

## Figures and Tables

**Table 1:** The core differences between metabolic compartments.

Metabolic Compartments	Core Features	Key Targets	Intervention Strategies	Refs.
Tumor Core Zone	Hypoxia, High Expression of CA IX, Glycolysis	CA IX/MCT4/LDHA	CA IX Inhibitor + LDHA Inhibitor	[97]
Invasion Front Zone	Rich in Oxygen, High Expression of CA XII, OXPHOS	CA XII/MCT1/PDHK1	MCT1 Inhibitor + PDHK1 Inhibitor	[87]
Metabolic Coupling Interface	Lactate Shuttle, Exosome Communication	Exosomal CA IX/ANXA2	ANXA2 Antibody + Exosome Capture Agent	[22,36]

## Abbreviations

CA IX	Carbonic Anhydrase IX.
CA XII	Carbonic Anhydrase XII.
TME	Tumor Microenvironment.
HIF	Hypoxia-Inducible Factor.
VHL	Von Hippel-Lindau (gene).
TCGA	The Cancer Genome Atlas.
EMT	Epithelial-Mesenchymal Transition.
ECM	Extracellular Matrix.
MMP	Matrix Metalloproteinase.
MT1-MMP	Membrane-Type Matrix Metalloproteinase 1.
FAK	Focal Adhesion Kinase.
CSCs	Cancer Stem Cells.
EGFR	Epidermal Growth Factor Receptor.
PI3K	Phosphatidylinositol 3-Kinase.
NHE1	Na^+^/H^+^ Exchanger 1.
TGF-β	Transforming Growth Factor Beta.
IGF-1	Insulin-like Growth Factor 1.
EV	Extracellular Vesicles.
MVBs	Multivesicular Bodies.
HUVECs	Human Umbilical Vein Endothelial Cells.
TAMs	Tumor-Associated Macrophages.
CCL8	C-C Motif Chemokine Ligand 8.
NF-κB	Nuclear Factor Kappa B.
PAAD	Pancreatic Adenocarcinoma.
PDAC	Pancreatic Ductal Adenocarcinoma.
AF	Auranofin.
NSCLC	Non-Small Cell Lung Cancer.
MCT1	Monocarboxylate Transporter 1.
MCT4	Monocarboxylate Transporter 4.
LDHA	Lactate Dehydrogenase A.
LDHB	Lactate Dehydrogenase B.
OXPHOS	Oxidative Phosphorylation.
5-FU	5-Fluorouracil.
CAR-T	Chimeric Antigen Receptor T-Cell Therapy.
BLA	Biologics License Application.
ADC	Antibody-Drug Conjugate.
SMDCs	Small Molecule-Drug Conjugates.
PET	Positron Emission Tomography.
P-gp	P-glycoprotein.
VEGF	Vascular Endothelial Growth Factor.
RCC	Renal Cell Carcinoma.
ccRCC	Clear Cell Renal Cell Carcinoma.
HCC	Hepatocellular Carcinoma.
HDI	Human Development Index.
HRE	Hypoxia Response Element.
TCA	Tricarboxylic Acid (Cycle).
ACC	Acetyl-CoA Carboxylase.
CAFs	Cancer-Associated Fibroblasts.
DFS	Disease-Free Survival.
OS	Overall Survival.
